# Comparison of Running Distance Variables and Body Load in Competitions Based on Their Results: A Full-Season Study of Professional Soccer Players

**DOI:** 10.3390/ijerph18042077

**Published:** 2021-02-20

**Authors:** Hadi Nobari, Rafael Oliveira, João Paulo Brito, Jorge Pérez-Gómez, Filipe Manuel Clemente, Luca Paolo Ardigò

**Affiliations:** 1 Department of Physical Education and Sports, University of Granada, 18010 Granada, Spain; 2 Department of Exercise Physiology, Faculty of Sport Sciences, University of Isfahan, Isfahan 81746-7344, Iran; 3 HEME Research Group, Faculty of Sport Sciences, University of Extremadura, 10003 Cáceres, Spain; jorgepg100@gmail.com; 4 Sports Science School of Rio Maior–Polytechnic Institute of Santarém, 2140-413 Rio Maior, Portugal; jbrito@esdrm.ipsantarem.pt; 5 Research Centre in Sport Sciences, Health Sciences and Human Development, 5001-801 Vila Real, Portugal; 6 Life Quality Research Centre, 2140-413 Rio Maior, Portugal; 7 Escola Superior Desporto e Lazer, Instituto Politécnico de Viana do Castelo, Rua Escola Industrial e Comercial de Nun’Álvares, 4900-347 Viana do Castelo, Portugal; filipe.clemente5@gmail.com; 8 Instituto de Telecomunicações, Delegação da Covilhã, 1049-001 Lisboa, Portugal; 9 Department of Neurosciences, Biomedicine and Movement Sciences, School of Exercise and Sport Science, University of Verona, 37131 Verona, Italy; luca.ardigo@univr.it

**Keywords:** association football, performance, load monitoring, high-speed running, match, match result

## Abstract

The aims of this study were to compare the external workload in win, draw and defeat matches and to compare first and second halves in the Iranian Premier League. Observations on individual match performance measures were undertaken on thirteen outfield players (age, 28.6 ± 2.7 years; height, 182.1 ± 8.6 cm; body mass, 75.3 ± 8.2 kg; BMI, 22.6 ± 0.7 kg/m^2^) competing in the Iranian Premier League. High-speed activities selected for analysis included total duration of matches, total distance, average speed, high-speed running distance, sprint distance, maximal speed and GPS-derived body load data. In general, there were higher workloads in win matches when compared with draw or defeat for all variables; higher workloads in the first halves of win and draw matches; higher total distance, high-speed running distance and body load in the second half in defeat matches. Specifically, lower average speed was found in matches with a win than with draw or defeat (*p* < 0.05). Sprint distance was higher in the first half of win than defeat matches and high-speed running distance was lower in draw than defeat matches (all, *p* < 0.05). In addition, first half presented higher values for all variables, regardless of the match result. Specifically, high-speed running distance was higher in the first half of matches with a win (*p* = 0.08) and total distance was higher in the first half of matches with a draw (*p* = 0.012). In conclusion, match result influences the external workload demands and must be considered in subsequent training sessions and matches.

## 1. Introduction

Quantification of training/match load represents an important procedure for adjusting training stimuli provided to players for the demands of the match [1,2]. The load quantification not only provide the researcher with clearer statistics, but also help coaches and sports scientists to gather more comprehensive performance data. Interest has grown in this area of study over the last decades as it enables sports scientists to identify the current demands placed on players in competition and apply data to training and testing protocols [3]. 

Previous studies have shown that internal and external load may vary throughout a season in different team sports [4,5,6,7,8,9,10]. Specifically, regarding variations in external imposed workloads, it has been shown that matches induce significant biochemical and neuromuscular responses related to fatigue [11] because of the high intensity actions demanded [12,13].

As such, matches are expected to have a significant effect on athletes’ weekly workloads. Studies have examined external workload imposed by official matches in an array of leagues around the world and despite a plethora of research, there are no criterion measure to distinguish physical performance in elite soccer matches has been identified but the total distance covered and particularly the large amount of high-speed running distance (HSRD) [14] and also the demands required by the accelerations and decelerations [15] seem to be useful metrics. For instance, the amount of HSRD accounts for approximately 8% of the total distance covered during match-play [16] and may be used as a valid measure of physical performance during soccer match-play. HSRD allows differentiating between different standards of play [17] and tactical role of players [18]. This measure is related to the overall success of the team [16,18]. Like all measures of sports performance, HSRD in soccer match-play are not stable properties but are subject to variation between successive matches [10,17].

Recently derived variables, such as the new player load or the new body load were proposed to investigate changes of direction along all the axes and can provide useful information for comprehending the external workload of training sessions and matches [12]. Recently, Nobari et al. [7] used body load (BL) in professional soccer players during a full-season and found that starter players present regularly higher values when compared to non-starters. The same authors stated that this variable is higher in matches. The use of BL along with distance variables will help to determine relevant information for coaches and their staff to better understand external workload imposed by matches. 

In addition, changes in the physical condition of the player [9,19] and contextual conditions [10] will lead to workload variations across the competitive season. Contextual factors such tactical formation, quality of opposition, and match stoppages may influence overall workloads during matches [8]. In light of the variability presented, where factors such as fitness are unlikely to change, it is important for practitioners to monitor the imposed demands on their own group of players. As a consequence of the variation that is likely to be mediated through both the inherent demands of the game and the individual’s ability to regulate their own activity, the variability in HSRD in soccer is likely to be relatively large [10]. When considering match scenarios, there are considerable inter-week variation in terms of determinant external load measures caused by the own dynamics of the match and its contextual factors [20]. The gained insights from a load profile which can and should be the foundation of team management will help coaches and staff to better adjust workload and periodization through the different phases of the season.

Despite the aforementioned research and, to the author’s knowledge, only one study has explored the potential relationship of match result (win, draw, defeat) on the external workload produced by professional soccer players [21], but without including running distance variables. Moreover, win matches produce higher external workload variables when compared with draw or defeat result [21] and produce relevant ramifications for subsequent training and competition. Notwithstanding, the accumulated fatigue during matches (differences between the first and the second half) produces a decreasing performance in total distance covered, HSRD or sprints [18,22]. A greater understanding of how this situational match result might be affecting external workload could help coaches and practitioners to make more informed decisions when prescribing subsequent training load. In addition, it could help to identify if there are certain matches results in the season when players might need additional support to cope with the demands (e.g., losing to a bottom-table team). 

Therefore, we expect to contribute to the literature by providing some reference values with regard to running distance and body load variables in won, defeated and drawn matches. Thus, the aims of this study were to compare the running distances variables and BL in win, draw and defeat matches in the Iranian Premier League (IPL). A comparison between first and second halves was also analysed for this study.

## 2. Materials and Methods 

### 2.1. Experimental Approach to the Problem

This study includes a professional team that participated in the highest level of professional football, the IPL (Persian Gulf Premier League and knockout tournament in this country). In this league, each team is allowed to use the Global Positioning System (GPS) to record the physical fitness statistics of its players. We analyse 33 matches where 16 wins, 14 draws and three defeats occurred. During each match, players were monitored by a GPS (Model: SPI High-Performance Unit HPU, GPSPORTS Systems Pty Ltd., Canberra, Australia) and the study variables were collected daily during the full-season (i.e., all training and matches). The two aims of this study were to describe and compare total duration of training session, total distance (TD), average speed (AvS), HSRD, total sprint distance (TSD), maximal speed (MS) and BL data collected in different match results and between first and second halves.

### 2.2. Participants

Thirteen professional soccer players (age, 28.6 ± 2.7 years; height, 182.1 ± 8.6 cm; body mass, 75.3 ± 8.2 kg; BMI, 22.6 ± 0.7 kg/m^2^, and total training and match time, 8935.2 ± 1214.7 min) participated in this study. All players of this team had at least a history of playing in the national youth team of Iran, or the highest level of the country’s soccer league from the youth category (over 10 years). The inclusion criterion required that players must participate in at least three training sessions each week. Also, player had to participate in three consecutive full matches. The exclusion criteria included: (i) players with prolonged injury or a lack of participation in training for at least two consecutive weeks (two players were removed based on this criterion); (ii) goal keepers were excluded from the study due to differences in training activities and workload in training and matches. The experimental approach and study design were presented to the players after which written consent was obtained from all players. The study followed the ethical guidelines of Helsinki Declaration for the study in Humans and was approved by the Ethics Committee of the University of Isfahan (IR.UI.REC.1399.064).

### 2.3. Monitoring External Load

#### GPS Receiver Specifications 

During the season, all workouts and match sessions were monitored using model SPI HPU GPS-based tracking systems for professional athletes, which offer 15 Hz position GPS and data source BL used a triaxial accelerometer. According to a previous study, this device has a high validity and reliability [23]. There were no reported adverse weather conditions to affect data collection. 

Data collection. Prior to the start of the match, belts were placed on the players’ shoulder and chest. After each cool down session at the end of the training, the belts were collected from the players. All belts were checked by the team’s GPS manager and then entered into the dock system to download the information, which was then stored on the computer with the Team AMS software. The data from each session was automatically deleted from the belt memory after download. Prior to the next session, the belts were placed in an electric charge station. The SPI IQ Absolutes were adjusted for GPS default zone throughout the season. Also, the personal characteristics (such as height and weight) of each player were entered in the software and each player registered a belt in his own name for use until the end of the season. The following variables were then selected: total duration of training session, TD, AvS, HSRD (18–23 km·h^−1^), TSD (>23 km·h^−1^), maximal speed (MS) and GPS-derived BL data.

### 2.4. Statistical Analysis

Data were analysed using the SPSS for Windows statistical software package version 22.0 (SPSS Inc., Chicago, IL, USA). Initially descriptive statistics were used to describe and characterize the sample. Shapiro-Wilk and Mauchly’s tests were used to verify the assumption normality and sphericity, respectively. Repeated measures ANOVA was used with Bonferroni post hoc, once variables obtained normal distribution (Shapiro-Wilk > 0.05) and it was used ANOVA Friedman and Mann-Whitney tests for the variables that not obtained normal, to compare different match results. Also, paired sample t-test was used to compare data from first half with second half according to the final result of the match. Results were significant with *p* ≤ 0.05. The effect-size (ES) statistic was calculated to determine the magnitude of effects by standardizing the coefficients according to the appropriate between subject’s standard deviation and was assessed using the following criteria: <0.2 = trivial, 0.2 to 0.6 = small effect, 0.6 to 1.2 = moderate effect, 1.2 to 2.0 = large effect and >2.0 = very large [24]. 

## 3. Results

Descriptive results and comparisons between wins, draws and defeats results of the variables studied are presented in Table 1.

Regarding total match differences based on results, there were significant differences between win and draw for match duration (ES = 0.36 [−0.43, 1.12]), between win and defeat (ES = 2.30 [1.25, 3.20]) and between draw and defeat for AvS (ES = 2.19 [1.16, 3.08]). In the 1st half, there were significant differences between win and draw for match duration (ES = 7.0 [4.45, 8.96]) and between win and defeat for sprint distance (ES = 1.15 [2.40, 5.38]). In the 2nd half, there were significant differences between win and defeat for HSRD (ES = 0.86 [0.09, 1.15]).

Descriptive results and comparisons between 1st and 2nd halves of the variables studied are presented in Table 2.

Regarding matches with a win there were significant differences between first half and second halves for HSRD (ES = 0.56 [−0.23, 1.34]). 

Regarding matches with a draw there were significant differences between first and second halves for match duration (ES = 0.61 [−0.18, 1,40]), TD (ES = 0.67 [−0.12, 1.46]), MS (ES = 0.12 [−0.65, 0.89]).

**Figure 1:**
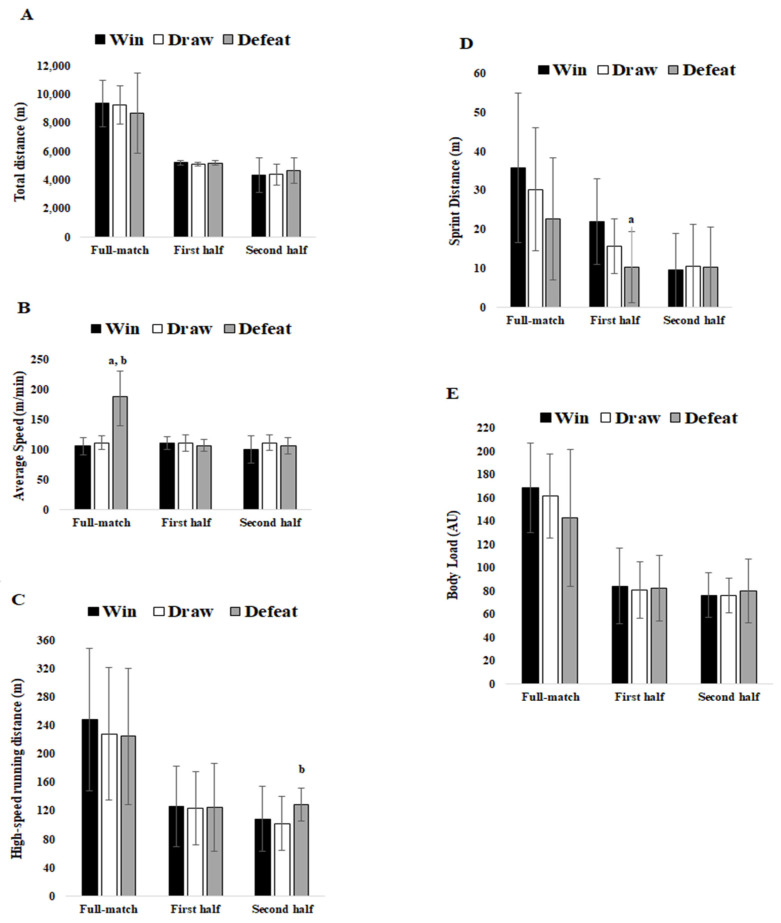
Comparisons between full, first and second halves for match results for (**A**) Total distance. (**B**) Average Speed. (**C**) High-speed Running Distance. (**D**) Sprint Distance. (**E**) Body Load. a denotes significant differences between win vs. defeat; a denotes difference between defeat vs. win; b denotes significant difference between defeat vs. draw. All (*p* < 0.05).

Regarding matches with defeat there were no significant differences. Descriptive results and comparisons between wins, draws and defeats for TD, AvS, HSRD, sprint distance and BL are also presented in Figure 1 for better clarity. 

## 4. Discussion

The aim of this study was to compare running distance variables and BL between matches with different results (win, draw and defeat) and also between first and second halves of matches. The main findings of the present study reflected that: (1) a win match outcome impacted significantly the duration thus in a win result the match duration is longer than in draws or defeat, and (2) with regard to the match outcome, higher AvS was covered in matches were a defeat occurred than those with win or draw results. 

The findings of the present study showed that the mean full-match duration in official matches is dependent on match outcome. We found that there were higher values in win than draw or defeat matches (win > draw > defeat). For instance, it was found that higher full duration in won matches is associated with higher total distance, HSRD, sprint distance and BL.

In the present study the results for TD covered during an official game are not dependent on match outcome. These findings are not corroborated by Smpokos et al. [25] who claim that a team cover significantly longer distance in won matches than in draw and lost matches (*p* < 0.001). According to the authors this could be explained by the observation that the team may regulate the physical efforts according to the specific demands of individual matches and periods of the game. The TD covered in the present study is, however, in the total playing time and in both halves of the game lower than those reported by previous studies [16,25,26,27,28,29]. In the present team studied, a plausible justification that can give grounds for similar TD regardless of the match results could be associated to the tactical system and strategy applied by coaches, even with more duration in won matches. 

Furthermore, when analysing match result, the results are similar to those reported by the study of elite German soccer players during the 2014–2015 from Andrzejewski et al. [30] that reported similar values for all position between different results with the exception of the forwards. This finding was also reported before by Lago et al. [31] in Spanish Premier League (SPL) players during the 2005–2006 season and more recently by Moalla et al. [28] in Stars League during 2013–2015. The present study cannot provide such finding once only thirteen players were included in the analysis and therefore, no comparison between player positions were conducted. 

When addressing to the halves of the game, some studies [32,33] have reported that a longer TD is covered during the first half of the match compared to the second half. In the present study there was a tendency on higher values in the first half with no statistically differences between halves of won and defeated matches. However, in matches with a draw, there was significant differences (*p* < 0.05). In opposition, Andrzejewski et al. [29] reported a slightly higher total distance covered in second half than the first half. Furthermore, the same study showed the same load patter regarding sprint distance. When analysing match results, the findings of the present study are similar to other study that found higher TD, HSRD and sprint distance when team was winning [28]. 

Previous studies have generally reported evidence of time-dependent reductions in the HSRD covered by players over the course of elite match play, from the first to second halves [34,35]. In fact, our study corroborated those findings in win and draw matches but not for defeat matches where higher values were found in the second half. In opposition, other studies showed that soccer players’ work rate was higher when losing than winning a match [28,36,37,38]. This can be attributed to the more offensive style of play when a team is in need of a goal than when they are not, and the players may, therefore, have a higher work rate when they are pursuing a goal [39]. It seems that when the team is losing, there are a tendency to players try to reach their maximal activity in order to win or draw the match [28,30]. Finally, it was found that values from the present study fall short when compared with Moalla et al. [28] study that observed more than 600 m of HSRD and 230 m of sprint distance or compared with Andrzejewski et al. [30] study that found more than 2000 m of HSRD, regardless of match result. 

The MS running in the present study was similar to the 28 km·h^−1^ reached by other study conducted in FA Premier League [33], lower than 31 km·h^−1^ conducted in SPL by Rey et al. [40], and lower than 31.9 km·h^−1^ conducted with Europa League soccer player by Andrzejewski, et al. [29]. However, it is important to notice that different GPS were used what could influence results. Even so, IPL players still need to improve to achieve the numbers presented by Europa League players. 

Andrzejewski, et al. [29] observed around 21 ± 3 m as the mean sprint distance covered regardless of the match result. However, the present study showed a much higher value specially in matches with a win or draw result. Only defeat matches presented similar values. These results could be attributed to the evolution of the game and the specificity of the IPL which not fully studied at this point. Also, it is important to acknowledge that Andrzejewski, et al. [29] analysed 147 soccer players and did not analyse the differences between match result. 

Regarding BL and to the best of the authors’ knowledge, no studies were conducted with the same variable. However, since BL is similar to player load, we observed that the pattern of the present study showed higher values in the first half which is in line with Reche-Soto et al. [21] study. The same study also found higher values for win matches than other results. Body load is obtained by calculating the sum of the accelerations in the three-movement axis, although some differences regarding this calculation are noticeable in the literature [41]. Despite accelerations were not analysed in this study, we speculate higher values in the first half. 

Some limitations should be addressed. Despite the study has a strong inclusion criterion in order to include a player for analysis, only thirteen players from a single team were included which is not large enough to make full generalizations. Another limitation of the present study is the unequal number of analysed matches with win (16), draw (14) and defeat (3) results that compromises the statistical power. Nevertheless, the present represent the actual training and competition environment from athletes.

Furthermore, there are other situational variables that could add some information regarding workload imposed by matches, such as location or quality of the opponents.

## 5. Conclusions

The study found higher workloads in win matches when compared with draw or defeat for all variables. In addition, it was found higher workloads in the first halves of win and draw matches, but in defeat matches, higher total distance, HSRD and BL was found in the second halves. The present results must be considered in subsequent training sessions and matches and will help to better periodization training load through the full-season.

For instance, in order to win or draw matches, it was found that TD covered should reach around 9263.4 ± 1350.8 to 9369.9 ± 1641.1 m; while BL should reach 168.6 ± 38.6 to 161.8 ± 36.3 au. In order to win matches HSRD and TDS should reach 247.9 ± 100.4 m and 35.9 ± 19.2 m, respectively, while AvS should be close to the 100 m/min. With the reported values, coaches and staff can prepare training sessions to achieve those values in some training sessions to simulate the demands obtained in won matches. Also, they can emphasize higher workloads in the first half of training sessions to achieve a better match result. 

## Figures and Tables

**Table 1:** Comparison of full match-day, first half and second half data between wins, draws and defeat per squad average, mean (SD) and CI, 95%.

Full-Match	Win (CI, 95%)	Draw (CI, 95%)	Defeat (CI, 95%)	CI, 95% (Win vs. Draw)	CI, 95% (Win vs. Defeat)	CI, 95% (Draw vs. Defeat)
Duration (min), n = 13	88.8 ± 11.9 * (81.6–95.9)	84.2 ± 13.7 (75.9–92.5)	82.1 ± 27.4 (65.5–98.6)	0.15 to 8.98	−12.94 to 26.32	−14.71 to 18.98
TD (m), n = 13	9369.9 ± 1641.1 (8378.2–10,361.6)	9263.4 ± 1350.8 (8477.1–10,109.6)	8673.6 ± 2828.8 (6964.1–10,383.0)	−698.03 to 851.08	−1474.13 to 2866.84	−1074.36 to 2314.02
AvS (m/min), n = 13	106.0 ± 14.8 ** (97.1–114.9)	111.2 ± 11.2 *** (104.4–117.9)	188.8 ± 48.8 (159.4–218.4)	−11.63 to 1.26	−119.04 to −46.74	−113.96 to −41.45
HSRD (m), n = 13	247.9 ± 100.4 (187.2–308.6)	228.0 ± 93.2 (171.6–284.3)	225.1 ± 95.7 (167.3–282.9)	−17.39 to 57.30	−57.44 to 103.04	−63.49 to 69.18
TSD (m), n = 12	35.9 ± 19.2 (23.7–48.1)	30.2 ± 15.8 20.1–40.2)	22.7 ± 15.6 (14.0–31.3)	−7.08 to 18.58	−4.80 to 31.30	−3.53 to 18.54
MS (km·h^−1^), n = 13	28.2 ± 2.2 (26.9–29.6)	28.8 ± 1.3 (28.0–29.6)	29.3 ± 1.5 (28.4–30.2)	−1.74 to 0.57	−2.66 to 0.51	−1.25 to 0.27
BL (au), n = 13	168.6 ± 38.6 (145.3–192.0)	161.8 ± 36.3 (139.8–183.8)	143.2 ± 58.8 (107.6–178.7)	−3.19 to 16.85	−17.81 to 68.73	−23.75 to 61.01
**1st half**	Win (CI, 95%)	Draw (CI, 95%)	Defeat (CI, 95%)	CI, 95% (win vs. draw)	CI, 95% (win vs. defeat)	CI, 95% (draw vs. defeat)
Duration (min), n = 10	47.2 ± 0.2 ** (46.7–47.7)	46.3 ± 1.2 (43.5–49.1)	48.6 ± 0.2 (48.1–49.0)	−2.58 to 4.40	−2.34 to −0.40	−5.76 to 1.20
TD (m), n = 10	5215.8 ± 156.1 (4862.7–5569.0)	5116.8 ± 108.9 (4870.5–5363.1)	5203.2 ± 151.0 (4861.7–5544.7)	−135.34 to 351.36	−137.53 to 162.70	−256.39 to 83.54
AvS (m/min), n = 10	110.5 ± 10.8 (102.8–118.3)	111.4 ± 13.5 (101.7–121.1)	107.1 ± 9.4 (100.4–113.8)	−8.31 to 6.59	−0.33 to 7.20	−3.95 to 12.54
HSRD (m), n = 10	126.6 ± 56.6 (86.1–167.1)	123.6 ± 51.9 (86.5–160.7)	124.6 ± 61.7 (80.5–168.7)	−15.67 to 21.68	−25.98 to 30.05	−30.70 to 28.77
TSD (m), n = 9	22.0 ± 11.1 ** (13.5–30.6)	15.7 ± 7.1 (10.2–21.6)	10.3 ± 9.2 (3.2–17.4)	−0.88 to 13.59	3.07 to 20.39	−3.45 to 14.19
MS (km·h^−1^), n = 10	28.9 ± 1.9 (27.5–30.3)	29.1 ± 1.4 (28.2–30.1)	29.1 ± 1.9 (27.7–30.5)	−1.88 to 1.36	−2.04 to 1.52	−1.12 to 1.12
BL (au), n = 10	88.4± 32.5 (65.2–111.7)	80.8 ± 24.4 (63.3–98.3)	82.1 ± 28.3 (61.8102.3)	−3.31 to 18.51	−1.51 to 14.20	−11.67 to 9.16
**2nd half**	Win (CI, 95%)	Draw (CI, 95%)	Defeat (CI, 95%)	CI, 95% (win vs. draw)	CI, 95% (win vs. defeat)	CI, 95% (draw vs. defeat)
Duration (min), n = 13	43.7 ± 7.6 (39.0–48.3)	39.8 ± 8.0 (35.0–44.7)	44.7 ± 10.1 (28.6–50.9)	−1.79 to 9.43	−11.07 to 8.95	−12.26 to 2.50
TD (m), n = 13	4340.6 ± 1207.3 (3611.0–5070.2)	4390.5 ± 741.4 (3942.5–4838.6)	4671.1 ± 883.3 (4137.3–5204.8)	−909.30 to 809.37	−1578.99 to 917.98	−898.63 to 337.54
AvS (m/min), n = 13	100.3 ± 23.3 (86.3–114.4)	111.6 ± 13.2 (103.6–119.6)	106.5 ± 14.1 (98.0–115.0)	−25.19 to 2.62	−21.84 to 9.48	−5.39 to 15.59
HSRD (m), n = 10	108.7 ± 46.0 (80.9–136.5)	102.4 ± 37.7 *** (79.6–125.2)	129.3 ± 23.3 (112.2–146.4)	−14.83 to 27.35	−52.08 to 10.81	−48.23 to −5.56
TSD (m), n = 11	16.9 ± 9.5 (10.5–23.3)	15.8 ± 10.6 (8.7–23.0)	15.6 ± 10.3 (8.7–22.5)	−8.95 to 10.99	−14.47 to 17.07	−13.20 to 13.76
MS (km·h^−1^), n = 13	28.5 ± 1.6 (27.5–29.4)	28.7 ± 1.3 (27.9–29.4)	29.6± 1.7 (28.6–30.6)	−1.25 to 0.83	−2.85 to 0.60	−2.09 to 0.26
BL (au), n = 13	76.3 ± 19.3 (64.6–88.0)	76.1 ± 15.2 (66.9–85.3)	80.0 ± 27.4 (63.5–96.6)	−14.48 to 14.93	−25.45 to 18.02	−18.42 to 10.53

au = arbitrary units; m = meters; TD = total distance; HSRD = high-speed running distance; TSD = total sprint distance; BL = body load; AvS = average speed; MS = Maximal speed; CI = confidence interval. * significant differences between win vs. draw, *p* < 0.05. ** significant differences between win vs. defeat, *p* < 0.05. *** significant differences between draw vs. defeat, *p* < 0.05.

**Table 2:** Comparison of first half and second half data for wins, draws and defeat per squad average, Mean (SD).

Variables	1st Half (CI, 95%)	2nd Half (CI, 95%)	*p*	CI, 95%
**Win**				
Duration (min), n = 13	45.1−7.9 (40.3–49.9)	43.7 ± 7.6 (39.0–48.3)	0.613	−4.64 to 7.54
TD (m), n = 13	5029.3 ± 954.0 (4452.9–5605.8)	4340.6 ± 1207.3 (3611.0–5070.2)	0.108	−174.83 to 1552.39
HSRD (m), n = 13	139.3 ± 59.4 (103.4–175.2)	108.7 ± 46.0 (80.9–136.5)	0.008 *	9.62 to 51.59
TSD (m), n = 13	18.2 ± 11.8 (11.0–25.3)	16.1 ± 8.9 (10.7–21.5)	0.380	−2.84 to 6.93
MS (km·h^−1^), n = 13	28.0 ± 4.4 (25.4–30.6)	28.5 ± 1.6 (27.4–29.4)	0.726	−3.41 to 2.44
BL (au), n = 13	92.3 ± 31.6 (73.2–111.4)	76.3 ± 19.3 (64.6–88.0)	0.129	−5.41 to 37.40
AvS (m/min), n = 13	111.8 ± 9.7 (105.9–117.7)	100.3 ± 23.3 (86.3–114.4)	0.063	−0.72 to 23.66
**Draw**				
Duration (min), n = 13	44.4 ± 6.5 (40.4–48.3)	39.8 ± 8.0 (35.0–44.7)	0.008 *	1.40 to 7.64
TD (m), n = 13	4902.9 ± 748.7 (4450.4–5355.3)	4390.5 ± 741.4 (3942.5–4838.6)	0.012 *	132.05 to 892.61
HSRD (m), n = 13	125.6 ± 63.2 (87.4–163.8)	102.4 ± 37.7 (79.6–125.2)	0.096	−4.80 to 51.13
TSD (m), n = 11	16.2 ± 7.6 (11.1–21.3)	15.8 ± 10.6 (8.7–23.0)	0.915	−6.80 to 7.50
MS (km·h^−1^), n = 13	28.9 ± 1.7 (27.9–29.9)	28.7 ± 1.6 (27.9–29.4)	0.499	−0.56 to 1.08
BL (au), n = 13	85.7 ± 23.9 (71.3–100.2)	76.1 ± 15.2 (66.9–85.3)	0.063	−0.61 to 19.86
AvS (m/min), n = 13	110.9 ± 12.0 (103.7–118.2)	111.6 ± 13.2 (103.6–119.6)	0.833	−7.58 to 6.22
**Defeat**				
Duration (min), n = 10	48.6 ± 0.6 (48.1–49.0)	47.1 ± 7.4 (41.8–52.4)	0.537	−3.73 to 6.69
TD (m), n = 10	5203.2 ± 477.4 (4861.7–5544.7)	4864.4 ± 481.4 (4520.0–5208.7)	0.054	−7.95 to 685.71
HSRD (m), n = 10	124.6 ± 61.7 (80.5–168.7)	130.6 ± 31.9 (107.8–153.5)	0.664	−36.68 to 24.53
TSD (m), n = 9	10.3 ± 9.2 (3.2–17.4)	11.8 ± 7.5 (6.1–17.6)	0.587	−7.79 to 4.72
MS (km·h^−1^), n = 10	29.1 ± 1.9 (27.7–30.5)	29.9 ± 1.8 (28.6–31.1)	0.307	−2.33 to 0.82
BL (au), n = 10	82.1 ± 28.3 (61.8–102.3)	77.9 ± 26.7 (58.8.97.0)	0.099	−0.96 to 9.27
AvS (m/min), n = 10	107.1 ± 9.4 (100.4–113.8)	105.2 ± 15.3 (94.2–116.1)	0.592	−5.96 to 9.84

au = arbitrary units; m=meters; TD = total distance; HSRD = high-speed running distance; TSD = total sprint distance; BL = body load; AvS = average speed; MS = Maximal speed. * denotes difference from 2nd half. all *p* < 0.05.

## Data Availability

The data presented in this study are available on request from the corresponding author.
